# Industry sponsorship bias in randomized controlled trials of digital cognitive behavioral therapy for insomnia: a meta-research study based on the 2023 Brazilian guidelines on the diagnosis and treatment of insomnia in adults

**DOI:** 10.3389/fneur.2025.1600767

**Published:** 2025-07-22

**Authors:** Viviane Akemi Kakazu, Marcia Assis, Andrea Bacelar, Andréia Gomes Bezerra, Giovanna Lira Rosa Ciutti, Silvia Gonçalves Conway, José Carlos Fernandes Galduróz, Luciano F. Drager, Mariana Pery Khoury, Ingrid Porto Araújo Leite, Ygor de Matos Luciano, Dalva Poyares, Sergio Tufik, Gabriel Natan Pires

**Affiliations:** ^1^Departamento de Psicobiologia, Universidade Federal de São Paulo, São Paulo, Brazil; ^2^Clínica do Sono de Curitiba, Hospital São Lucas, Curitiba, Brazil; ^3^Clínica Bacelar—Neuro e Sono, Rio de Janeiro, Brazil; ^4^Instituto de Psiquiatria (IPq), Universidade de São Paulo, São Paulo, Brazil; ^5^Departamento de Otoneurologia, Universidade de São Paulo, São Paulo, Brazil; ^6^AkasA—Formação e Conhecimento, São Paulo, Brazil; ^7^Unidades de Hipertensão, Instituto do Coração (InCor) e Disciplina de Nefrologia, Universidade de São Paulo, São Paulo, Brazil; ^8^Faculdade de Medicina da Universidade Santo Amaro (UNISA), São Paulo, Brazil; ^9^Instituto do Sono, São Paulo, Brazil; ^10^Hospital Israelita Albert Einstein, São Paulo, Brazil; ^11^Faculdade Israelita de Ciências da Saúde Albert Einstein, Hospital Israelita Albert Einstein, São Paulo, Brazil

**Keywords:** sleep, industry sponsorship bias, cognitive behavioral therapy, systematic review, meta-research

## Abstract

**Background:**

Industry sponsorship bias refers to the tendency of a study, most likely clinical trials, to produce results that favor the sponsor’s interest. It is especially relevant in cases in which a study is funded by companies or organizations with a commercial interest in the product or technology being evaluated. Digital Cognitive Behavioral Therapy for Insomnia (dCBT-I) is a widely used nonpharmacological treatment, and research in this area is often funded by organizations that have a commercial interest in this treatment. This study aimed to assess whether industry sponsorship bias in dCBT-I trials is associated with more favorable outcomes.

**Methods:**

This study was based on the sample of randomized controlled trials (RCTs) included at the “2023 Brazilian Guidelines on the Diagnosis and Treatment of Insomnia in Adults.” This guideline was based on a systematic review conducted in the PubMed and Web of Science databases, searching for randomized controlled trials (RCTs) on dCBT-I. Inclusion criteria included 1. Studies performed with adults with non-comorbid insomnia, diagnosed using the International Classification of Sleep Disorders 3rd edition (ICSD), the Diagnostic and Statistical Manual of Mental Disorders, 5th edition (DSM-5), or with moderate to severe insomnia symptoms evaluated using the Insomnia Severity Index (ISI) or the Athens Insomnia Scale (AIS). 2. dCBT-I as intervention. 3. Other forms of CBT-I or negative control groups (no treatment, placebo, waiting list, or minimal intervention) as comparators, and 4. ISI as main outcome. For each included study, sponsorship bias was analyzed in a 5-points scale, considering the role of the sponsor (from “no funding received” to “all author authors are affiliated to the company developing the dCBT-I”). Immediate post-treatment ISI data was extracted for both intervention and control groups in each included study, and the between-groups Cohen’s d effect size was calculated for each included study. Methodological quality in each included RCT was evaluated using the van Tulder scale. Statistical analyses were performed to investigate possible associations between the levels of sponsorship bias and the results of the studies.

**Results:**

Twenty-eight analyses of RCTs were included. Interventions such as SHUTi (Sleep Healthy Using the Internet) (39.28%) and Online CBT-I (28.57%) were the most common, with comparators such as minimal intervention (50%) and waiting list (32.14%). There was a significant association between the risk of sponsorship bias and open access publication [X^2^(1)=5.250; *p* = 0.022], as well as between the risk of sponsorship bias and lower levels of methodological quality [X^2^(1)=4.861; *p* = 0.027]. There was no correlation between risk of bias levels and Insomnia Severity Index (ISI) mean scores (the main indicator of outcomes) in the control and experimental groups. These results suggest that the risk of sponsorship bias may impact the methodological quality of studies and compliance with established standards.

**Conclusion:**

A greater risk of sponsorship bias was associated with lower methodological quality articles and open access publication.

## 1 Introduction

Research biases refer to systematic errors that occur during the stages of preparation, conduct and analysis of a research, which compromise the veracity of the results and conclusions (1). According to the University of Oxford’s Catalogue of Bias, “industry sponsorship bias” refers to “the tendency of a scientific study to support the interests of the study’s financial sponsor” (1). This term refers to distortions in the design, performance, interpretation of findings and/or publication of a study that favor the commercial interests of its sponsor (2). As examples, this can occur when the research question is biased toward results favorable to the sponsor, non-representative populations are chosen, an article is not published because the data is unfavorable to its sponsor, or there is partial and selective reporting of results, among other reasons.

It is common for companies to sponsor research, and this is not necessarily a problem. On the contrary: privately funded research can often make a significant contribution to the advancement of science, and in some areas this might be the only source of funding available (2). Even some Nobel Prizes have been awarded for research conducted solely in the private sector, such as studies on G-protein coupled receptors by Robert Lefkowitz and Brian Kobilka, from the company ConfometRx (Nobel Prize in Medicine—2012) (3) and the development of blue LED by Shuji Nakamura, from Nichia Corporation (Nobel Prize in Physics–2014) (4). Reputable universities all over the world receive significant amount of investments for research from private companies, and the pharmaceutical industry is one of the largest investors (5). Nonprofit agencies such as the Wellcome Trust, the U. S. Food and Drug Administration (FDA), and the Gates Foundations encourage this type of collaboration (2), which might be advantageous for both parties, provided that the best scientific and methodological standards are observed.

Unfortunately, these standards are not always observed, and in some cases are severely disregarded. One example of this related was the growing evidence in the 1950s showing that smoking was linked to lung disease (6). This led cigarette manufacturers and the tobacco industry to fund research that said otherwise (6). This strategy manipulated public opinion and health policies for an entire generation. As a consequence, the World Health Organization continues to reject partnerships with the tobacco industry, stating that there is a clear conflict of interest (7).

In the field of sleep medicine, sponsored research occurs primarily in respect of clinical trials of drugs, and the development and validation of new sleep technologies and treatments (8). One of these is Cognitive Behavioral Therapy for Insomnia (CBT-I), a non-pharmacological treatment that is currently considered the gold standard for the treatment for chronic insomnia (9). It was initially developed with no, or very limited, investment from non-academic parties. However, with the development of digital therapeutics versions of CBT-I (dCBT-I), industry-sponsored research became more frequent (9, 10). A recent meta-epidemiological study showed that the number of publications on non-pharmacological therapies for insomnia has increased significantly since the 2000s, and that the number of RCTs of dCBT-I treatments has surpassed the number of RCTs of pharmacological interventions for insomnia, which possibly results from the increased number of apps being developed by healthtech and startup companies in this area, and their desire to generate evidence of their effectiveness (11).

Companies often have a particular interest in sponsoring and publishing research on their own products, both to prove their efficacy and safety to health regulatory agencies, and to better position themselves in the market. However, it remains unclear whether the commercial interests of companies developing dCBT-I influence the results of the research. Therefore, the main objective of this study was to evaluate whether the risk of sponsorship bias was related to more positive outcomes in RCTs of dCBT-I.

## 2 Methods

This is a meta-research study based on a secondary analysis of the data used to formulate the “2023 Guidelines on the Diagnosis and Treatment of Insomnia in Adults,” published by the Brazilian Sleep Association, which included a systematic review aimed to identify and assess studies that investigated the effects of CBT-I in adults with chronic insomnia and without comorbidities (12). A detailed description of the methods used in this systematic review were published in the parenting study (12). The PRISMA (Preferred Reporting Items for Systematic Reviews and Meta-Analyses) guidelines were followed to ensure clarity and transparency in the reporting of data. The completed PRISMA checklist is available as Supplementary material. Some PRISMA-recommended information, such as detailed search strategies and protocol and registration details, are more fully described in the original parent study, and are duly acknowledged in the checklist (12). The aspects that are relevant to the current study are described below.

### 2.1 Search strategy and study selection process

The search strategy and study selection process was completely based on the procedures implemented in the parenting study, with no modifications. Systematic searches were performed in the PubMed and Web of Science databases, using a search strategy divided into two domains: one for insomnia and the other for CBT-I. Gray literature and other forms of secondary search strategies were not implemented. The retrieved records were imported into the Covidence^®^ platform, where deduplication was automatically performed.

The studies were evaluated in a two-phase process (12): The first phase included the evaluation of titles and abstracts, while the second included the evaluation of the full text. In both phases, each study was evaluated by two of six independent reviewers. Any conflicts about the inclusion of a study were resolved by a third reviewer (GNP). The inclusion criteria applied in the original systematic review considered both pharmacological and non-pharmacological interventions for insomnia eligible (12). In the current analyses, the final sample of articles was further narrowed to include only studies about dCBT-I. The following inclusion criteria were applied:

- *Abstract and language*
- *Inclusion*: Only articles with abstracts published in English or Portuguese.
- *Exclusion*: Articles with no abstract or published in a language other than Portuguese and English.
- *Type of articles*
- *Inclusion*: Randomized controlled trials (RCTs), including cross-over trials.
- *Exclusion*: Any other study design.
- *Population*
- *Inclusion*: All of the following: 1. Adults with insomnia disorder, diagnosed according to the International Classification of Sleep Disorders, 3rd edition (ICSD-3), the Diagnostic and Statistical Manual of Mental Disorders, 5th edition (DSM-5), or compatible diagnostic manuals; or adults with moderate to severe insomnia symptoms according to the Insomnia Severity Index (ISI) or the Athens Insomnia Scale (AIS). 2. Studies in adults (considering adults the age group between 18 and 65 years). 3. Non-comorbid insomnia, evaluated at a population level.
- *Exclusion*: Any of the following: Studies in which insomnia was diagnosed or evaluated based on subjective reports of insomnia, assessed using instruments other than those aforementioned, studies including mild insomnia evaluated through the ISI or AIS, populations composed of insomnia comorbid with other conditions, and studies in an age group other than adults.
- *Intervention*
- *Inclusion*: CBT-I delivered digitally.
- *Exclusion*: Any non-digital form of CBT-I.
- *Control group*
- *Inclusion*: No treatment, in-person or another modality of CBT-I, placebo, or minimal intervention (including but not limited to sleep hygiene or sleep education).
- *Exclusion*: Any of the following: studies that lacked a control group, before-and-after study designs, group subjected to pharmacological intervention, control group subjected to concurrent ineligible intervention.
- *Outcome*
- *Inclusion*: Insomnia symptoms measured by the Insomnia Severity Index (ISI).
- *Exclusion*: Studies that did not report ISI as an outcome.

### 2.2 Data extraction and evaluation of industry sponsorship bias

The following information was extracted from each selected article:

*Publication details:* The model of publication, business model (open access or subscription based), and the name of the journal in which the study was published.

- *ISI scores after treatment:* This data was extracted from both the dCBT-I and the control groups for each article. Only immediate post-treatment data was considered. Data was extracted as mean and standard deviation (SD), or converted into it if presented in other formats.
- *App/site evaluated:* The name of the app or website used for the CBT intervention was collected when it was mentioned in the article. When it was not mentioned, the interventions were categorized as “Online CBT-I” when the intervention was telemedicine, meaning the patient was attended by a therapist in real-time via cell phone or computer; “In-person CBT-I,” used to describe situations where the patient was attended by a therapist in person; and “web-based Delivery,” when the information about CBT-I treatment was available online and the patient accessed it without a therapist’s intervention.
- *Company participation and conflicts of interest:* The authors’ association with the companies developing the dCBT-I was evaluated. This includes both the direct affiliation of authors with the company developing the dCBT-I intervention, or any declared sponsorship or funding of any form being received by the authors from the company developing the dCBT-I. The number and percentage of authors associated with the companies developing the dCBT-I interventions was calculated.
- *Sponsorship Bias Risk Rating:* Industry sponsorship was rated according to the following scale:
  0. *Very low risk:* No author is affiliated with the company developing the dCBT-I and there is no mention of sponsorship or funding being received from the company.
  1. *Low risk:* No author is affiliated with the company developing the dCBT-I, but there is mention of sponsorship or funding from this company.
  2. *Moderate risk:* At least one author is affiliated with the company developing the dCBT-I.
  3. *High risk:* The majority of the authors are affiliated with the company developing the dCBT-I.
  4. *Very high risk:* All authors are affiliated with the company developing the dCBT-I.

For some analyses, sponsorship bias levels 1–4 were grouped together, to compose a group of “possible bias,” in opposition to a bias level of 0, which was considered as considered as “very low bias.” This dichotomization allows separating those studies that received some sponsorship from a dCBT-I developer, from those that received no sponsorship.

### 2.3 Assessment of methodological quality

The methodological quality of each study was assessed using the van Tulder scale (13), which is an 11 item scale focused on internal validity and the quality of reporting in RCTs. These 11 items evaluate four main domains of methodological biases: selection, performance, detection, and attrition bias. In the analyses in this review, we considered that studies with a score ≤6 were categorized as being of a “lower methodological quality” and those with a score ≥7 of a “higher methodological quality.” The data for this analysis represents a secondary analysis of previously published data (11).

### 2.4 Data synthesis and analysis

The unit of analysis in this study is the comparison (or experiment) between a dCBT-I intervention and its respective control group. Some articles may include more than one dCBT-I experiment, so the number of analyses may not match the number of articles. These analyses are reported separately when applicable.

Data analysis was conducted at two levels. First, a descriptive analysis was performed to summarize the characteristics of the sample in the dCBT-I studies. Subsequently, an inferential analysis was performed to investigate possible associations between the levels of sponsorship bias and the results of the studies. For these analyses, three ISI-derived outcomes were considered: the average ISI score in the dCBT-I group, the average ISI score in the control group, and the effect size on the ISI score, calculated using Cohen’s d. For each included study, the effect sizes were extracted between-groups. Positive effect size values indicate that the experimental group had lower ISI scores, i.e., better response to treatment.

The following statistical tests were used in the analysis: Spearman’s test was performed to verify whether there is a correlation between the risk of sponsorship bias levels and the ISI scores. The effect of sponsorship bias on ISI scores was analyzed using Kruskal-Wallis test (when considering the five-level sponsorship bias scale) and using independent *t*-tests (for the dichotomized sponsorship bias results). Chi-square tests were performed to evaluate the associations between sponsorship bias and publication modality (open access or subscription-based models) and with each question on the van Tulder methodological quality scale. Continuous variables are presented as mean ± SD, and categorical data as frequencies and percentages. All analyses were performed using JAMOVI software, with statistical significance set at *p* < 0.05.

## 3 Results

### 3.1 Descriptive analysis of the sample

**Figure 1:**
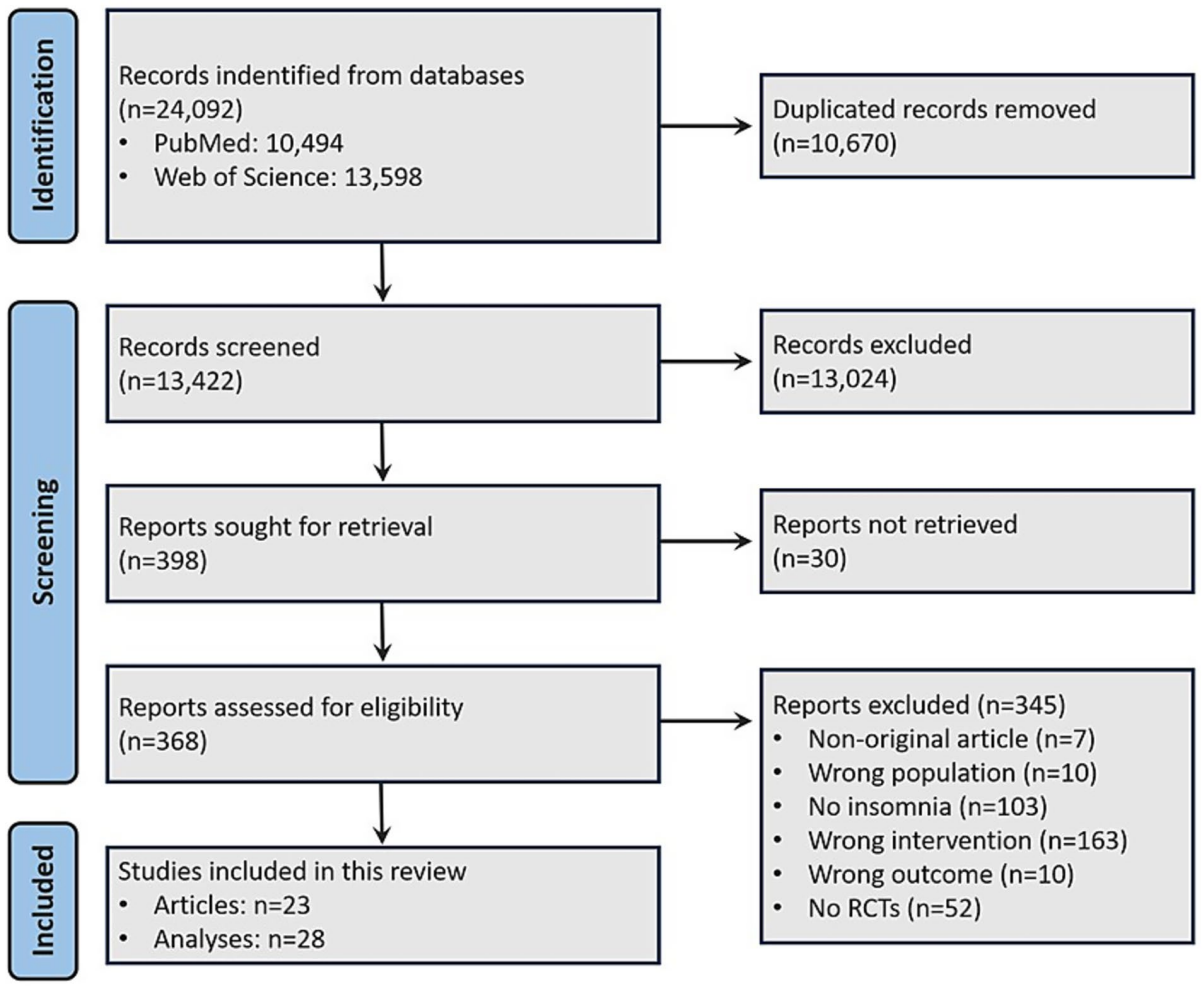
Flowchart of article identification and inclusion process.

The search strategy yielded 13,422 non-duplicated records. After screening and eligibility analyses, 23 articles were considered eligible and were included in the final sample, comprising a total of 28 analyses (Table 1). The article selection process is described in Figure 1.

**Table 1:** Descriptions and characteristics of the included studies.

Article	Analysis (n)	Authors (n)	Authors company affiliation (n)	Authors with exclusive company affiliation (n)	Mention of sponsorship	SB level	Sex	App/site evaluated	Comparator	Intervention length
Bernstein et al. (55)	1	12	12	10	Present	4	Both	Go! to Sleepª	No treatment/waiting list	6 weeks
Blom et al. (56)	1	5	0	0	Absent	0	Both	Web-based delivery	Minimal intervention (sleep hygiene)	8 weeks
Faaland et al. (57)	3	13	0	0	Present	1	Both	SHUTi	Minimal intervention (sleep hygiene)	9–11 weeks
Hagatun et al. (58)	1	10	0	0	Absent	0	Both	SHUTi	Minimal intervention (sleep hygiene)	9 weeks
Holmqvist et al. (36)	1	3	0	0	Absent	0	Both	Web-based delivery	Online CBT-i	6 weeks
Horsch et al. (19)	1	8	0	0	Present	1	Both	Sleepcare	No treatment/waiting list	6 weeks
Kallestad et al. (38)	1	9	0	0	Absent	0	Both	SHUTi	In-person CBT-I	6–9 weeks
Kjorstad et al. (59)	1	11	0	0	Absent	0	Both	SHUTi	Minimal intervention (sleep hygiene)	6 weeks
Kuhn et al. (60)	1	10	0	0	Absent	0	Both	Coach Mobile App	No treatment/waiting list	6 weeks
Lancee et al. (61)	1	4	0	0	Absent	0	Both	Online CBT-i	No treatment/waiting list	6 weeks
Lancee et al. (37)	2	5	0	0	Absent	0	Both	Online CBT-i	No treatment/waiting list	6 weeks
Lien et al. (62)	1	10	1	1	Present	2	Both	SHUTi	Minimal intervention (sleep hygiene)	9 weeks
Lopez et al. (63)	1	10	0	0	Absent	0	Both	Online CBT-i	Minimal intervention (sleep hygiene)	12 weeks
Lorenz et al. (64)	1	5	0	0	Present	1	Both	Online CBT-i	No treatment/waiting list	6 weeks
Rajabi Maid et al. (65)	1	9	0	0	Absent	0	Both	Theory-Based CBT App	Minimal intervention (sleep hygiene)	6 weeks
Ritterband et al. (66)	1	7	0	0	Present	1	Both	SHUTi	Minimal intervention (sleep hygiene)	9 weeks
Van der Zweerde et al. (67)	1	6	0	0	Absent	0	Both	i-Sleep	No treatment/waiting list	5 weeks
Vedaa et al. (68)	1	12	1	1	Present	2	Both	SHUTi	Minimal intervention (sleep hygiene)	9 weeks
Vincent et al. (69)	1	2	0	0	Absent	0	Both	Online CBT-i	No treatment/waiting list	6 weeks
Vincent et al. (70)	2	2	0	0	Absent	0	Both	Online CBT-i	No treatment/waiting list and In-person CBT-I	5 weeks
Yang et al. (71)	1	21	0	0	Absent	0	Both	We chat Mini-Program	Minimal intervention (sleep hygiene)	1 week
Zhou et al. (72)	2	6	0	0	Present	1	Women	SHUTi	Placebo/sham and Minimal intervention (sleep hygiene)	6–9 weeks
Zhang et al. (73)	1	6	0	0	Present	1	Both	Resleep	Minimal intervention (sleep hygiene)	6 weeks

n, frequency/number of events; SB, sponsorship bias; SHUTi, Sleep Healthy Using the Internet.

Most studies and analyses were classified as having a very low risk of sponsorship bias (studies: *k* = 14, 60.9%, analyses: *k* = 16, 57.1%). Some risk of sponsorship bias was observed in nine studies (39.1%) and 12 analyses (42.9%), but only one study with a single analysis (4.3%) was rated as having a very high risk of sponsorship bias (Table 1).

The mean ISI score in the control group (*n* = 2,859) was approximately 15.04 (±4.89), while in the experimental group (*n* = 2,696) it was 10.75 ± 5.06. The mean effect size was 1.06 ± 1.66. Most studies included both sexes/genders, with only one article focusing exclusively on a female sample. Regarding the intervention time, 16 studies had a duration until 6 weeks, and 12 studies had a duration more than 6 weeks.

A total of 10 different dCBT-I interventions were identified, mostly in the form of smartphone applications (“apps”). The most common were SHUTi (Sleep Healthy Using the Internet), present in 39.28% (*n* = 11) of the analyses, and online CBT-I that was not further specified, with 28.57% (*n* = 8). In terms of comparators, 50% (*n* = 14) of the studies used minimal interventions (such as sleep hygiene), 32.14% (*n* = 9) used a control group without treatment or a waiting list, 10.71% (*n* = 3) used face-to-face CBT-I therapy, and 3.5% (*n* = 1) used placebo/sham interventions.

### 3.2 Sponsorship bias and ISI scores

**Figure 2:**
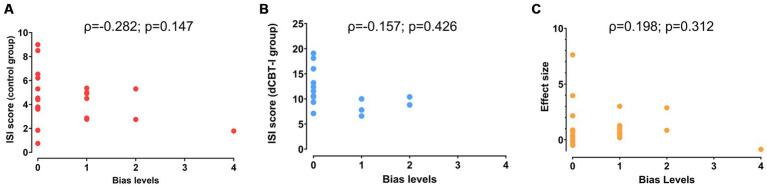
Correlations between risk of sponsorship bias levels and ISI score. Correlations of sponsorship bias were performed in three different contexts: with ISI score in the control group **(A)**, with ISI score in the experimental group **(B)** and with ISI effect size **(C)**.

The Spearman correlation test indicated no correlation of the sponsorship bias with the mean ISI score in the control group (*ρ* = −0.282; *p* = 0.147), and the mean ISI in the dCBT-I group (ρ = −0.157; *p* = 0.426), or in ISI score effect size (ρ = 0.198; *p* = 0.312; Figure 2).

**Figure 3:**
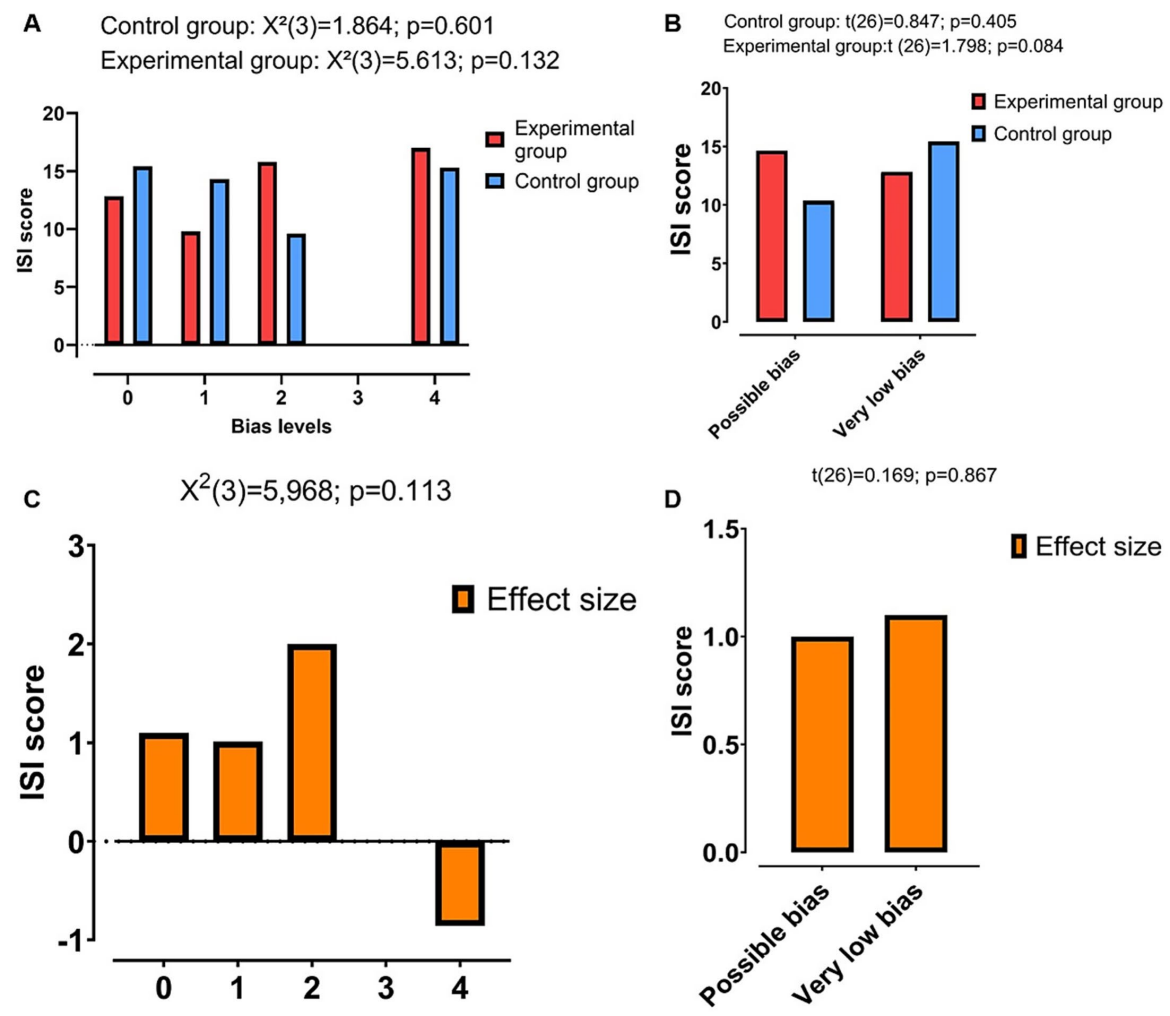
Comparison between the ISI score and sponsorship bias between the experimental and control groups, and effect size. Comparison made considering all levels of bias and Insomnia Severity Index (ISI) score **(A)**, only “possible bias” and “Very low bias” and ISI score **(B)**, all levels of bias and Effect size **(C)**, and only “possible bias” and “Very low bias” and Effect size **(D)**.

The levels of sponsorship bias had no significant effect on the mean ISI in either the control group [X^2^_(3)_=1.864; *p* = 0.601], the experimental group [X^2^_(3)_=5.613; *p* = 0.132] (Figure 3A), or in effect size [X^2^_(3)_=5,968; *p* = 0.113] (Figure 3C). The results remained unchanged in the analyses considering the dichotomization of sponsorship bias with no statistically significant difference in the ISI score between the groups, in the control [*t*_(26)_ = 0.847; *p* = 0.405], in the dCBT-I group [*t*_(26)_ = 1.798; *p* = 0.084] (Figure 3B), and in respect of effect size [*t*_(26)_ = 0.169; *p* = 0.867] (Figure 3D).

### 3.3 Sponsorship bias and methodological quality

**Figure 4:**
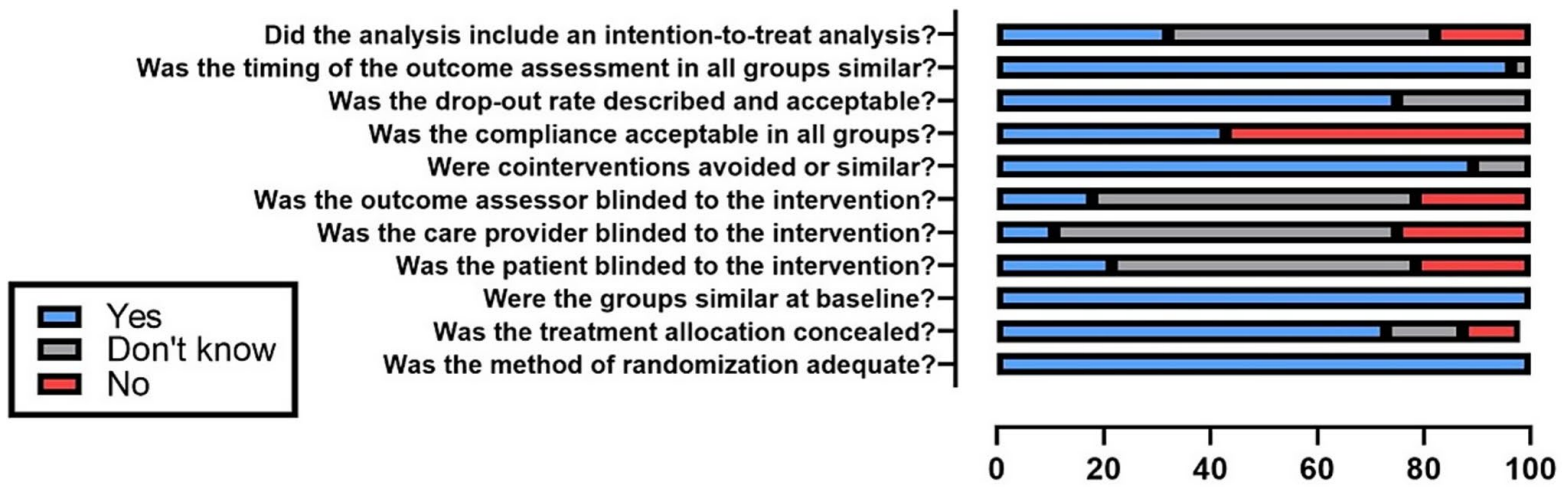
Methodological quality assessment considering the entire sample.

The mean compliance rate on the van Tulder scale across the 28 analysis was 65 ± 11.2% (Figure 4). The highest compliance rates were observed in item #3 (similar groups at baseline, *n* = 28, 100%), #10 (similar timing of outcome assessment, *n* = 27, 93.43%) and #7 (no co-interventions, *n* = 25, 89.29%). The lowest rates were recorded in items #5 (blinding of caregivers, *n* = 3, 10.71%) and #6 (outcome assessor blinded, *n* = 5, 17.83%). Considering these results, 12 studies (42.86%) were considered as having lower methodological quality (a score ≤6), while 16 (57.14%) had higher methodological quality (a score ≥7).

In the item-by-item analysis of the van Tulder scale, the only statistically significant association was observed between sponsorship bias and the item “Was the dropout rate described and acceptable?,” indicating that articles with sponsorship bias tend to report dropout rates less frequently (Table 2).

**Table 2:** Methodological quality assessment.

Criteria for the methodological quality	Very low bias (*n* = 16)			Possible bias (*n* = 12)				
	Yes	No	Do not know	Yes	No	Do not know	X^2^	*p*-value
1. Was the method of randomization adequate?	16 (100.00%)	0 (0.00%)	0 (0.00%)	12 (100.00%)	0 (0.00%)	0 (0.00%)	-	-
2. Was the treatment allocation concealed?	10 (62.5%)	3 (18.75%)	3 (18.75%)	11 (91.66%)	1 (8.33%)	0 (0.00%)	3.549	0.170
3. Were the groups similar at baseline regarding the most important prognostic indicators?	16 (100.00%)	0 (0.00%)	0 (0.00%)	12 (100.00%)	0 (0.00%)	0 (0.00%)	-	-
4. Was the patient blinded to the intervention?	3 (18.75%)	11 (68.75%)	2 (12.5%)	3 (25%)	5 (41.66%)	4 (33.33%)	2.394	0.302
5. Was the care provider blinded to the intervention?	3 (18.75%)	11 (68.75%)	2 (12.5%)	0 (0.00%)	7 (58.33%)	5 (41.66%)	4.699	0.095
6. Was the outcome assessor blinded to the intervention?	5 (34.25%)	8 (50%)	3 (18.75%)	0 (0.00%)	9 (75%)	3 (25%)	4.581	0.101
7. Were cointerventions avoided or similar?	14 (87.50%)	2 (12.5%)	0 (0.00%)	11 (91.66%)	1 (8.33%)	0 (0.00%)	0.124	0.724
8. Was the compliance acceptable in all groups?	7 (43.75%)	0 (0.00%)	9 (56.25%)	5 (41.66%)	0 (0.00%)	7 (58.33%)	0.012	0.912
9. Was the drop-out rate described and acceptable?	15 (93.75%)	1 (3.57%)	0 (0.00%)	6 (50%)	6 (50%)	0 (0.00%)	7.000	0.008*
10. Was the timing of the outcome assessment in all groups similar?	16 (100.00%)	0 (0.00%)	0 (0.00%)	11 (91.66%)	1 (8.33%)	0 (0.00%)	1.383	0.240
11. Did the analysis include an intention-to-treat analysis?	7 (47.75%)	8 (50%)	1 (6.25%)	2 (16.66%)	6 (50%)	4 (33.33%)	4.381	0.112

**Figure 5:**
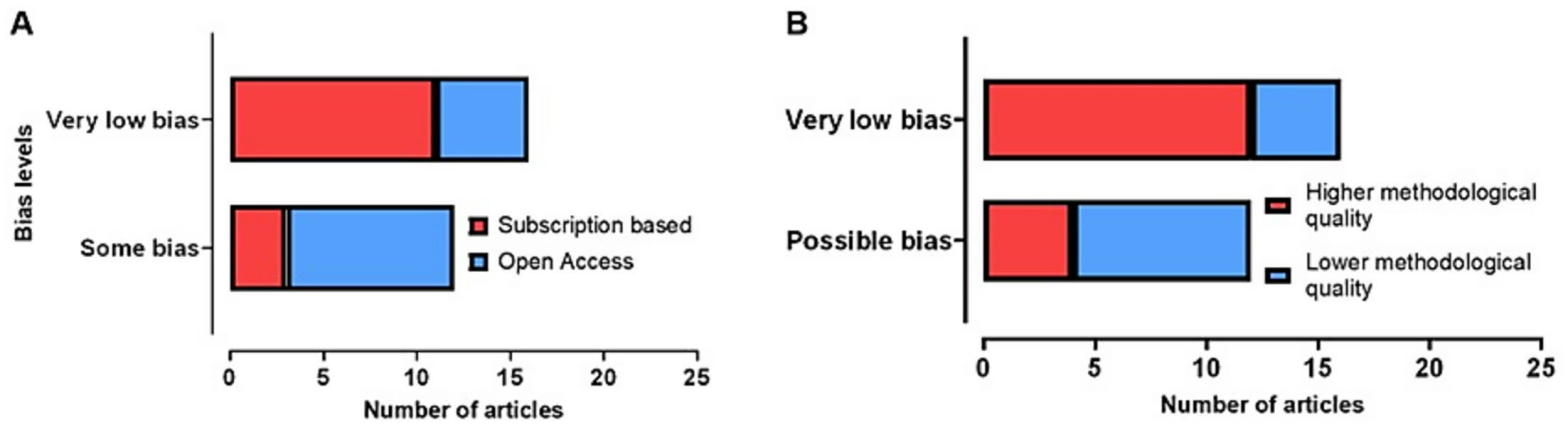
Comparison between the sponsorship bias levels (“Very low bias” and “Possible bias”). **(A)** Sponsorship bias levels were compared with the publication model (Subscription based and Open Access). **(B)** Sponsorship bias levels were compared with methodological quality (Higher and Lower methodological quality).

There was a significant association between sponsorship bias and overall methodological quality [X^2^(1)=4.861; *p* = 0.027], suggesting that studies with possible sponsorship bias are associated with lower methodological rigor (Figure 5B).

### 3.4 Sponsorship bias and publication models

A significant association between sponsorship bias and the publication model was observed [X^2^(1)=5.250; *p* = 0.022], indicating a tendency for articles with sponsorship bias to be published in open access. As depicted in Figure 5A, it can be seen that the articles with very low sponsorship bias are associated with an increased proportion of articles published in subscription-based journals, while articles with “possible bias” are more often published in open access journals. There was no association between methodological quality and publication models [X^2^(1)=0.583; *p* = 0.445].

### 3.5 Publication models and ISI scores

**Figure 6:**
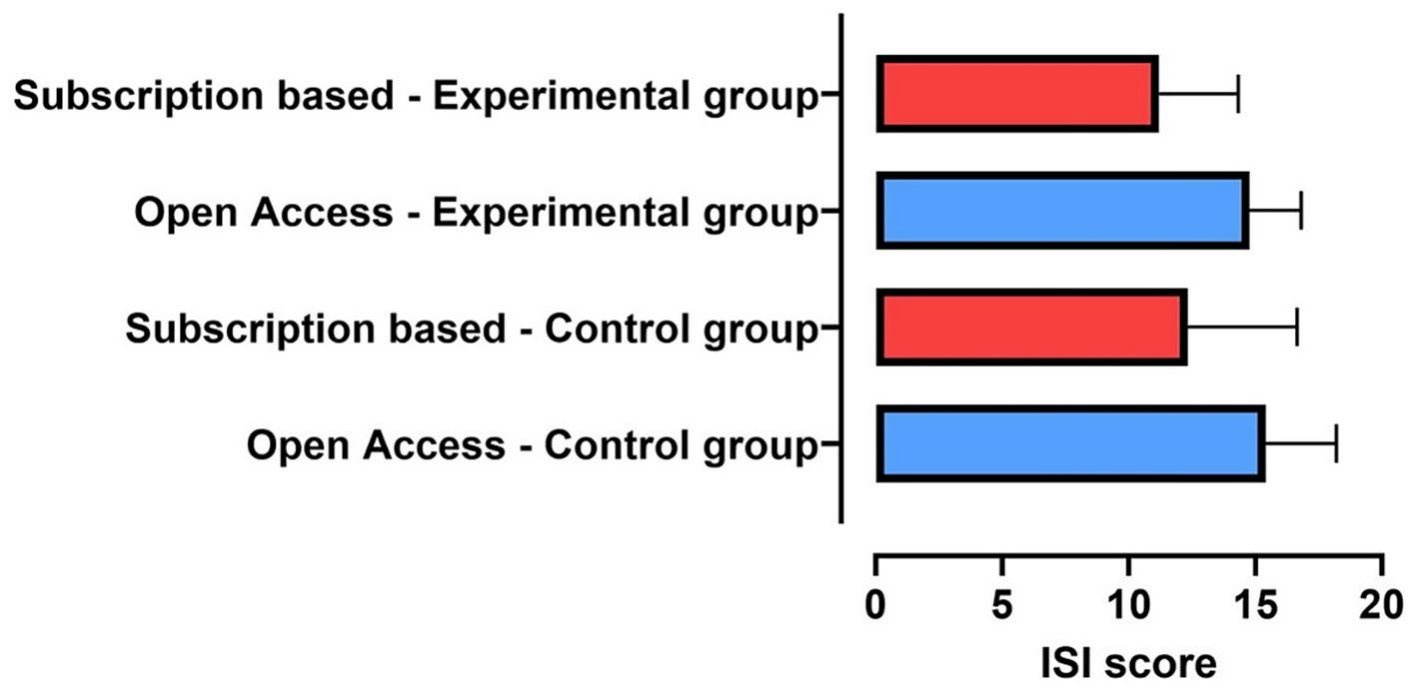
Comparisons between ISI score and the publication model (subscription-based and open access) in the control and experimental groups.

Figure 6 shows that the ISI values for the control group and dCBT-I group in open access publications are slightly higher than the values in the subscription-based publications. However, an independent *t*-test showed that this relationship was only apparent. No significant association was observed between publication models and the ISI score of the control group [*t*(26)=0.693; *p* = 0.494], and of the dCBT-I group [*t*(26)=0.789; *p* = 0.437].

## 4 Discussion

ISI is the most frequently used tool to evaluate response to treatment in RCTs about insomnia. In our study, we selected investigations that evaluated the impact of dCBT-I on this well-accepted metric for evaluating insomnia severity. Therefore, our results demonstrate that the risk of sponsorship bias is not related to inflated, overestimated or otherwise increased outcome reporting. However, analyses related to methodological quality and publication models yielded interesting results. Articles with possible sponsorship bias were more often published in open access journals and were associated with a lower methodological quality. Taking all this together, it can be concluded that the risk of sponsorship bias does not affect the main outcome results in RCTs about insomnia, but affects the methods implemented to achieve these results, and how these results are reported.

When several statin manufacturers were putting their products on the market, Bero et al. reviewed the relevant literature to understand whether statins were being favored when compared to other drugs (14). As a result, “favorable” analyses of statins were observed when the newer drug outperformed the competitor, “inconclusive” when the results were not statistically significant, and “unfavorable” if the older competitor drug was superior. These data demonstrate the behavior of companies sponsoring statin studies to favor their own product by overriding unfavorable results. A Cochrane systematic review found that industry-sponsored studies of drugs and medical devices usually report more favorable results. These results were not fully explained by an increased risk of bias among sponsored studies, as only one of many evaluation items seemed to be associated with sponsored studies (bias from blinding). Thus, the review concluded that there was a case of sponsorship bias, but that it is not conditionally related to overall risk of bias, as frequently measured as a matter of methodological robustness (15). This data contrasts with ours, as we observed no effect of sponsorship on the final results, but an important association with methodological quality. Another review also revealed that non-pharmacological treatments for insomnia appear to have lower methodological quality in general (11). This highlights the need to rigorously monitor methodological standards in sponsored research, especially of non-pharmacological treatments, ensuring that scientific integrity is maintained.

It was observed that the risk of sponsorship bias was associated with open access publications. This can be explained by at least two reasons. The first is that funding companies have a strong interest in ensuring that their research results are widely disseminated and accessible to the public. The second is that unsponsored research might not have sufficient funding to cover the costs of open access, which are considerably higher than those publications in traditional subscription-based journals. However, the argument of being accessible is not always synonymous with quality. In the literature on plastic surgery (16) and physiotherapy (17), studies have shown that the methodological quality was higher in subscription-based journals than in open access journals. Open access publications allow for greater visibility and reach, facilitating the communication of scientific findings to the public and the academic community without subscription barriers. This transparency not only increases the visibility of the research but also encourages the rapid adoption of the results in clinical practice or in the development of new products. Even so, it is important to question whether the works are methodologically robust, as well as whether open access publications are being favored because they are seen as an easier publication route.

Sponsorship bias was also associated with low methodological quality in the van Tulder scale, as the proportion of studies categorized within the “lower methodological quality” range is higher among the RCTs in the “possible bias” group. In individual items within the van Tulder scale, the only item that was different among groups was related to the reporting of dropouts, which was more frequent among RCTs categorized as with “very low bias.” The data distribution for the item related to the inclusion of intention-to-treat analysis (ITT) also calls attention, although not statistically significant. While almost half of the studies in the “very low bias” group include ITT analyses, only 16% of the studies in the “possible bias” group reports it. Reporting dropouts and performing ITT analyses are related items, as both are methodologically associated. It means, the performance of ITT analysis depends on proper control and reporting of dropouts. This is especially relevant when considered that adherence to treatment and attrition along dCBT-I programs are major challenges (18), and that patients undergoing digital therapeutics for insomnia are more likely to dropout from treatment at premature stages (18, 19).

The number of studies evaluating dCBT-I studies has been increasing considerably (11), which has led not only the Brazilian guidelines of insomnia but many other guidelines and consensuses by many sleep societies worldwide to recommend digital interventions for the treatment of insomnia at different levels (12, 20–24). This reflects the multiple meta-analysis currently available attesting the efficacy of dCBT-I in treating insomnia symptoms (18, 25–35). However, the differential efficacy between of digital and face-to-face CBTi is still uncertain. A few systematic review have performed analyses inferring that in-person CBT-I (either face-to-face or through telehealth) is more effective than dCBT-I (18, 27, 29, 33). However, these conclusions are always based on indirect inferences, usually by means of comparing the effect sizes of different CBT-I modalities vs. waiting list controls in independent studies. The best way to directly compare the effects of F2F and digital intervention would be to have RCTs directly comparing these two modalities, but this is a rather uncommon research design. To the best of our knowledge, only three RCTs provide some comparisons between digital and in-person CBT-I (36–38), none of them performed or sponsored by dCBT-I manufacturers. Although there might be others, the fact is that the vast majority of RCTs approaching dCBT-I are performed in comparison with inactive control groups (waiting list, placebo interventions, sleep hygiene, etc.), rather than in comparison to face-to-face or telehealth CBT-I.

The methods used by dCBT-I developers to validate their interventions has already been a matter of concerns, as they may often employ substandard research practices (8, 10, 11, 20). The term “digital exceptionalism” has been employed to describe the practices of digital therapeutics companies when trying to validate their products without using gold-standard practices (10). As a parallel, RCTs for the validation of new pharmacological interventions for insomnia often compare new hypnotic medication to at least one first line treatment (most frequently zolpidem) are common (39–41). However, this practice still has not been implemented for dCBT-I, as RCTs comparing them to either face-to-face or telehealth CBT-I are scarce.

The most often argued benefits of dCBT-I include being more accessible, affordable, and having a better cost-effective relationship, seem to be valid (20, 42–44). Recent studies have argued that the low costs associated with dCBT-I might justify its implementation into primary care and public health setting, and that its potential for scalability makes it a relevant tool for expanding access to evidence-based insomnia treatment, particularly in underserved areas (45–48). As an example, a study conducted within the Veterans Health Administration (VHA) (48) in the United States evaluated the feasibility of implementing dCBT-I—specifically the SHUTi program—in primary care clinics. The program was offered as a low-cost, highly accessible alternative for insomnia treatment. The results suggested that dCBT-I can be effectively and sustainably integrated into large-scale health systems, offering a feasible and effective alternative to traditional insomnia treatments.

Regardless of that, the lack of studies directly comparing in-person and digital CBT-I and the consequent low certainty of evidence regarding the comparable effects of these modalities leads us to support the common recommendation that assisted or in-person CBT-I (including face-to-face and telehealth) should be preferred (18, 26) over dCBT-I. Digital formats should be considered when assisted CBT-I is not possible for any cause, including patient’s preferences, limited availability, costs, among others.

### 4.1 Limitations

Some considerations are necessary for a proper interpretation of our findings. Most importantly, this is a secondary analysis of the data used in the development of the Brazilian guidelines on the diagnosis and treatment of insomnia in adults (12), which may limit the scope of this study. Therefore, the results herein reported are circumscribed to the referred guidelines insomnia, and the applicability of these findings to other guidelines and to the overall field of digital CBT-I is an indirect inference. The most important limitation related to being a secondary analysis of these guidelines regards the inclusion of articles dealing only with clinical diagnosis of insomnia (as per ICSD-3 or DSM-5) or with moderate to severe insomnia symptoms (as per ISI or AIS), as well as restricting outcome assessment to the ISI. Therefore, studies in which insomnia diagnosis was exclusively based on self-report or did not contain moderate to severe insomnia symptoms, were not included. Although the ISI is the most commonly used symptom assessment tool in insomnia research, there are other questionnaires and scales widely used for this purpose, including the Bergen Insomnia Scale (BIS) and the Sleep Condition Indicator (SCI). The specific non-inclusion of SCI leads into a secondary inclusion bias: this is the insomnia evaluation tool of choice for most RCTs evaluating Sleepio, one of the pioneers and most known available dCBT-I intervention (49, 50). It means that not including SCI conditionally excluded Sleepio from our sample of articles. The level of sponsorship bias on these studies would likely to be high on our 1–5 scale, as a significant number of these RCTs have authors affiliated to Sleepio’s manufacturer (51–53). But the effects of including SCI and the consequent inclusion of RCTs evaluating Sleepio on the results of our study are uncertain. In any case, these same criteria were applied to the parenting study, meaning that the results remain valid if interpreted as a secondary analysis of the Brazilian insomnia guidelines.

Also, only RCTs were included, as this methodological design is considered the gold standard of methodological rigor for intervention studies. However, studies about the effects of dCBT-I with other methodological designs are numerous, many of which highlight real-world evidence (RWE) approaches (54), which resemble traditional type four post-marketing surveillance trials. As RWE studies are less expensive and more subjected to methodological biases (especially inclusion and attrition bias), the association of outcome reporting with sponsorship bias might have been different if non-randomized approaches (including RWE) were included. Finally, the decision to adhere to the sample of studies used in the Brazilian insomnia guidelines conditionally subjected the current study to methodological aspects implemented in the parenting study. This includes not performing gray literature screening or other forms of secondary searches. A different methodological approach, eventually considering other insomnia evaluation tools (such as the SCI or including mild insomnia symptoms at the ISI), research designs other than RCTs (such as RWE), and a broader literature search (including gray literature) would have resulted in a larger sample of articles to be evaluated. This would have both negative aspects (such as considering research design associated with a lower level of evidence), and positive aspects (such as a bigger and more diverse set of studies to be evaluated). Such a broader approach is encouraged for future research.

## 5 Conclusion

No correlation was found between the risk of industry sponsorship bias and the ISI score. However, a significant association was observed between the risk of sponsorship bias and both the publication modality and methodological quality, with articles with possible sponsorship bias being significantly associated with open access publication and lower quality.

## Data Availability

The datasets presented in this article are not readily available because this dataset is private and available upon request. Requests to access the datasets should be directed to gabriel.pires@unifesp.br.
